# Automated chemical synthesis. Part 3: Temperature control systems

**DOI:** 10.1155/S1463924683000279

**Published:** 1983

**Authors:** Daniel F. Chodosh, Sidney H. Levinson, John L. Weber, Kenneth Kamholz, Charles E. Berkoff

**Affiliations:** Smith Kline & French Laboratories Inc. Preclinical Research & Development 1500 Spring Garden Street Philadelphia Pennsylvania 19101 USA


Journal of Automatic Chemistry, Volume 5, Number 2 (April–June 1983), pages 103–107

### Automated chemical synthesis. Part 3: Temperature control systems

**Daniel F. Chodosh, Sidney H. Levinson, John L. Weber, Kenneth Kamholz and Charles E. Berkoff**

*Smith Kline & French Laboratories, Inc., Preclinical Research & Development, 1500 Spring Garden Street, Philadelphia, Pennsylvania 19101, USA*

Research efforts have been directed towards constructing a fully automated microcomputer-controlled bench-scale 'batch-type' chemical reactor [1–6]. This fully automated reactor can execute repetitive experiments on an approximately 50 ml scale, providing important chemical reaction data during early stages of process development research programmes. The precision and accuracy of the reagent/solvent delivery systems, chemical analysis systems and temperature-control systems are, of course, of fundamental concern. Fail-safe operation is an equally important design consideration if the apparatus is to be used in an unattended operation mode. Particular emphasis has been placed on both self-checking features (for example flow monitors verify proper solution movement through Teflon delivery lines) and fail-safe recovery (engineered into hardware and designed into the automation software). A key hardware element in the fail-safe construction, the opto-isolator interface, has been described previously [1 and 3–6]. With fail-safe operation considerations foremost in mind, a temperature-control system which is controlled by a Digital Equipment Corporation MINC LSI 11/2 computer system and its associated interfaces has been designed and constructed.

To effect temperature control in the chemical reactor vessel the opposing actions of systems that remove and add heat to the vessel contents must be balanced. Since the intended application of the apparatus entails repetitive, statistically directed experimentation [7–9], the temperature-control systems must be capable of reaching the designated reaction temperature set-point as quickly as possible (to maximize ramping), thereby minimizing the overall experimentation time. The heating and cooling systems must respond quickly to computer control signals and must have sufficient heating/cooling capacity to effectively respond to and control thermal anomalies of chemical reactions (for instance endotherms, exotherms).

### Cooling systems

The reactor vessel has been fitted with an internal glass coil (4·0 mm O.D. tubing, 35·0 mm coil diameter, 3·5 turns) with plastic glass-to-teflon connectors on the reactor head. The coils are spaced (about 3·0 mm between turns) to permit good mixing characterics in the vessel. The coolant (MeOH : $H_2O$, 1 : 2, v/v) is provided by a modified Neslab Model RTE-4 Refrigerated Bath Circulator (Portsmouth, NH, USA). The bath provides a fixed pressure of coolant which is manually adjusted via a valve on the bath and remains constant throughout the course of experimentation. The temperature of the bath is monitored by the computer via a thermistor (Thermomemtrics, Series CSP, Edison, NJ, USA) which replaces the thermometer supplied by the manufacturer. The flow of coolant through the reactor vessel coil is moderated by a needle valve (Whitey, No. SS-3LRF4, Cleveland, OH, USA) which is under computer control. The system is shown diagramatically in figure 1.

As received from the factory, the Neslab unit is equipped with three manually operated toggle switches: a master power ON/OFF switch, a compressor ON/OFF switch (mounted on the front panel) and a programmer accessory ON/OFF switch (mounted on the rear panel). A plug is provided for signal inputs used in external temperature tracking. To operate the unit, the user selects a nominal temperature on the front dial, powers the unit turning the compressor on and waits for temperature equilibrium to establish. A self-contained proportional control circuit modulates the heater circuit (800 W) to balance the cooling of the compressor (nominally 400 W). The selection of the Neslab unit was in part based upon the large capacity of its heating and cooling units proportional to the bath reservoir capacity (5 l). For set-point temperatures greater than 45°C, the compressor must be left off. If an external programmer is to be used, the operator moves the programmer accessory switch to 'on' once equilibirum has been established. As an external voltage is then applied via the programmer accessory receptacle the bath will change by 1°C/6·5 mV from the manually selected nominal dial temperature. The direction of the temperature change is determined by the polarity of the applied voltage. The most simple automation scheme would be to use a D/A (digital-to-analogue) converter from the computer to provide the requisite external voltage to the programmer input circuit; in order, however, to provide more intimate computer control with appropriate safeguards, hardware modifications to the unit were necessary.

The interfacing of the compressor on/off function was quite straightforward. A TTL-driven solid-state relay (Crydon, #27F324, Address: El Segundo, CA, USA) was installed in parallel with the manual compressor control switch. The manual switch is left in the off position—deferring compressor control to the computer via a single TTL on/off signal. Two important operational enhancements are achieved. First, if the desired bath set-point temperature is greater than 45°C, the system can be operated without manual intervention: the computer system can turn the compressor on only when the temperature nears the new designated set-point. In this manner, the full 800 W of heating capacity are used to reach the new set-point in half the time that would be required were the compressor running (800 W heating minus 400 W cooling). Second, should there be a failure in the system, as determined by the computer through software or a failure of computer system itself, the interfaces provided will automatically select a default

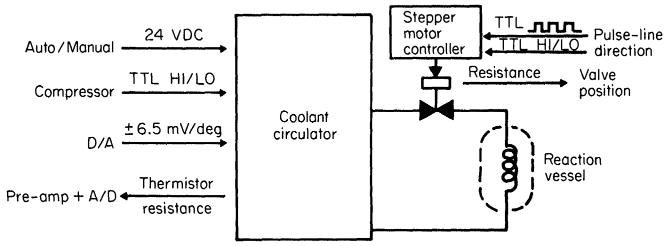

*Figure 1. Diagram of the cooling system showing the interface signals.*

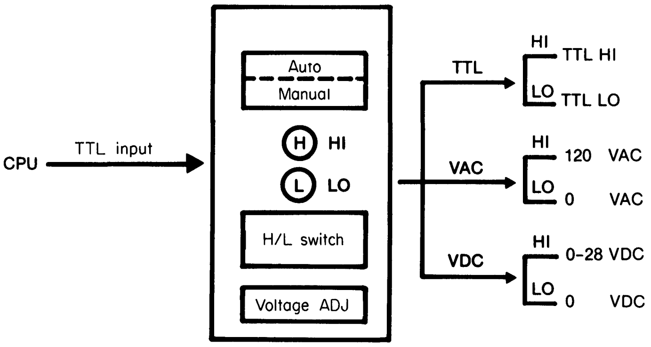

*Figure 2. Block diagram of the opto-isolator interface (single channel).*

signal and send that signal to the compressor unit. This latter feature derives from the operation of an opto-isolator interface through which the computer drives the compressor relay (see figure 2).

The bath start-up procedure has been described above. Integrity of this procedure has been retained to allow later applications that might require it. In order to automate the function of the programmer accessory toggle switch, an additional manual switch and a computer-driven relay were added. The circuit is shown in figure 3. A manually operated DPDT switch selects the operation mode: 'computer' or 'non-computer'. In 'non-computer' mode, the circuit is open at position A and closed at position B, defeating the relay. The programmer accessory on/off switch operates as originally intended. To operate in 'computer' mode, the operation switch is set to 'computer' and the programmer accessory switch to 'on'. This effectively toggles the programmer accessory switch via the relay (24 VDC). In the unenergized state the relay is normally closed ('nc') at position C and normally open ('no') at position D, and the unit operates as if the programmer accessory switch were off. By applying a 24 VDC signal via the opto-isolator interface, the circuit opens at C while closing at D. In this manner the external voltage supplied at pins 3 and 4 of the Jones receptacle are available for temperature offsetting. In the event of failure, the opto-isolator interface will, by the default switch setting for the indicated 24 VDC line, de-energize the relay and the bath will automatically return to the nominal temperature originally set on the front dial.

The interfacing signal complement also includes a thermistor which provides a temperature-dependent resistance signal. This signal is converted by the computer interfacing circuitry to a voltage and made available to the computational algorithms via an analogue-to-digital (A/D) converter. Once the computer measures the current equilibrium temperature and calculates a new temperature set-point, the offset voltage corresponding to the required temperature change is then applied to pins 3 and 4 on the programmer accessory receptacle via a digital-to-analogue (D/A) interface. The computer interface operates in bipolar mode with a range of +2·56V to −2·56V, allowing the computer to change the bath set-point temperature 39·4°C (2·56 V/6·5 mV) above or below the 'dial' temperature. The direction of change is determined by the polarity of the applied voltage. Control is stepwise, however, due to the resolution of the D/A interface (12 bit, 1·25 mV per step). Control is thereby limited to discrete temperature steps of 0·19° (1·25 mV/6·5 mV). A voltage-halving circuit could be added to increase the resolution of control at the expense of the dynamic range. Alternatively, a 14- or 16-bit D/A interface could be used—the higher resolution would decrease the step size.

In this semi-distributed control scheme the coolant temperature can be reliably modulated by the computer system. Temperature changes on the order of $\pm 1°C\,min^{-1}$ are observed, and the overall experimentation software is designed to take the system's slow response into consideration. To achieve rapid control of heat removal, a needle valve controlled by a high torque stepper motor is interposed. An on-board clock and direction select switch are included, allowing manual intervention. The flow of coolant is modulated by the computer via this assembly. The direction select line establishes the direction of valve movement (open/close) and each pulse applied to the pulse-line steps the motor by one position. Both signals are made available from a 16-bit parallel output computer interface (via the opto-isolator interface). The valve position is sensed by the computer through a 1000 Ω variable potentiometer (Clarostat #62JA, Dover, NH, USA) mechanically linked to the valve stem. This signal is accessed by the computer through an A/D converter as described earlier. This fast response system now augments the much slower response of the bath temperature controller, to afford excellent dynamic control of heat removal from the vessel.

### Heating system

To maximize the heat input (maximum temperature ramping), while minimizing any local heating effects, an immersion heating element (nominally, 60 W) and a cladding heater (40 W) are used. Within the limits of the available internal space of the reactor vessel, the immersion element (Watlow, #E1J40, St. Louis, MO, USA) has been fitted with a grooved stainless-steel sheath (SS316) to increase its effective surface area—thereby minimizing localized solution heating effects. (The effective surface area is about 104 $mm^2$.) The thermal conductivity of the sheath alleviates any problems associated with heat latency effects. The exterior of the glass vessel is coated with a thermal element (ACE Glass, INSTATHERM, Vineland, NJ, USA), which helps to insulate the vessel for better thermal stability and also provides even heating over a large surface area (about 440 $mm^2$). The systems, operating together, will provide approximately 100 W to the reaction vessel and its contents.

The interfacing circuit (see figure 5), permits precise dynamic computer control of the heaters and has reliable fail-safe features. The system design requires two independent d.c. power supplies, 96 VDC, 0·5 A (immersion) and 10 VDC, 4 A (cladding). These supplies are powered through independent channels of the opto-isolator interface. In the event of a failure, the interface defaults in order to shut off each power supply and the heating stops. The heaters are controlled by selectively powering either d.c. supply (or both) then supplying a voltage (D/A interface) to the proportionating control circuit. Figure 6 explains this circuit. A computational algorithm determines the percentage power to be applied to the heater(s) (0%–100%). A corresponding voltage signal (0 V–10 V) is directed to the voltage-to-frequency converter via a D/A interface (unipolar mode, 0 V to + 10·26 V). The frequency output of the V/F device has been set to range linearly from 0 Hz (0 V and 10 V, respectively) through external timing resistors (see figure 7). This frequency signal is attenuated by a factor of 10 and then used to trigger a retriggerable monostable vibrator ('one-shot') whose time period has been set to 1 ms. This scheme reshapes the D/A voltage into a linearly proportioned on/off TTL signal train. As shown, the output state of the 'one-shot' is always high at 10 V input to the V/F device. As the voltage decreases, the 'one-shot' smoothly proportionates between high and low states; at 5 V

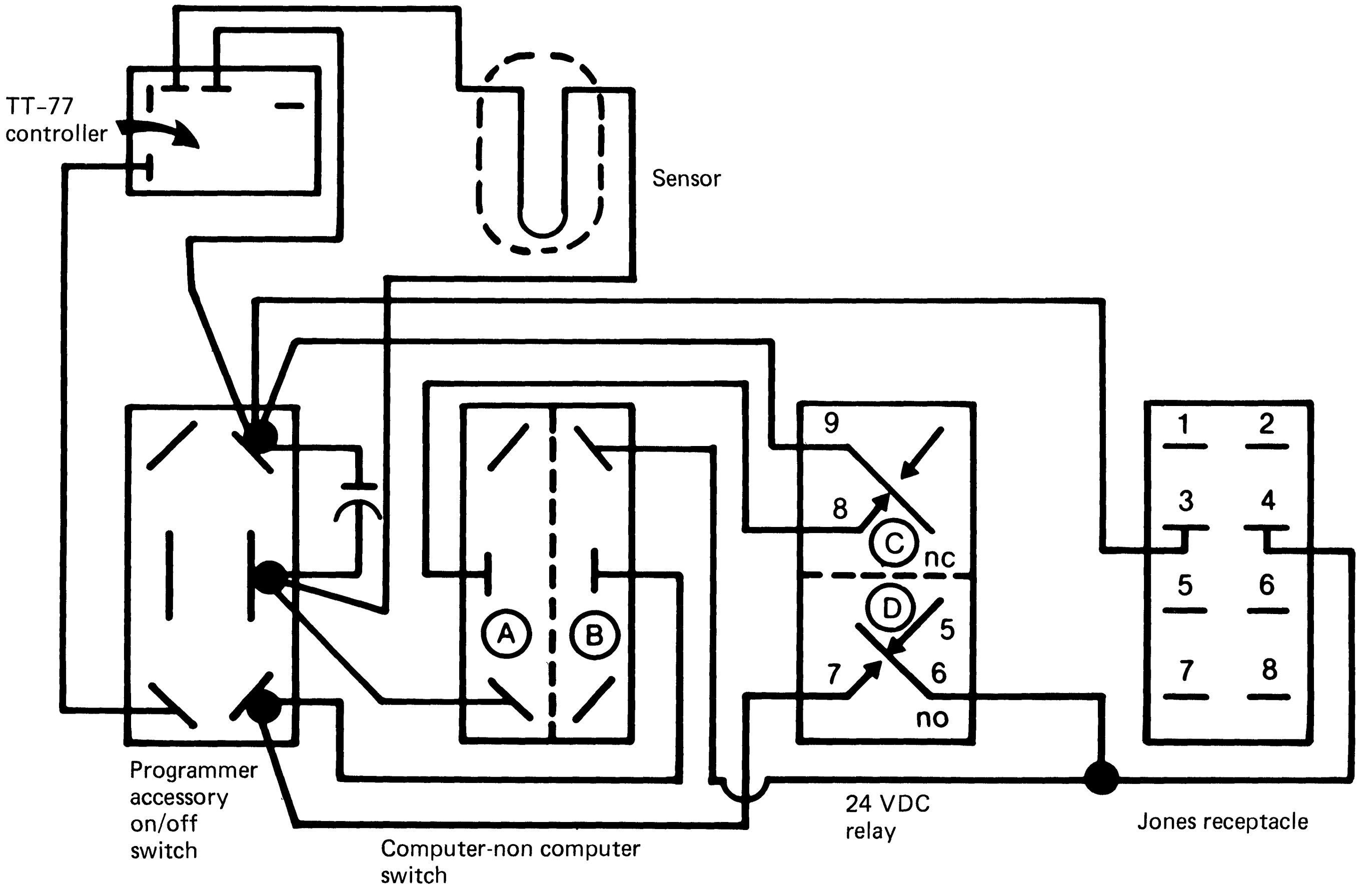

*Figure 3. Schematic diagram of the recirculating bath with modifications for interfacing to the computer system.*

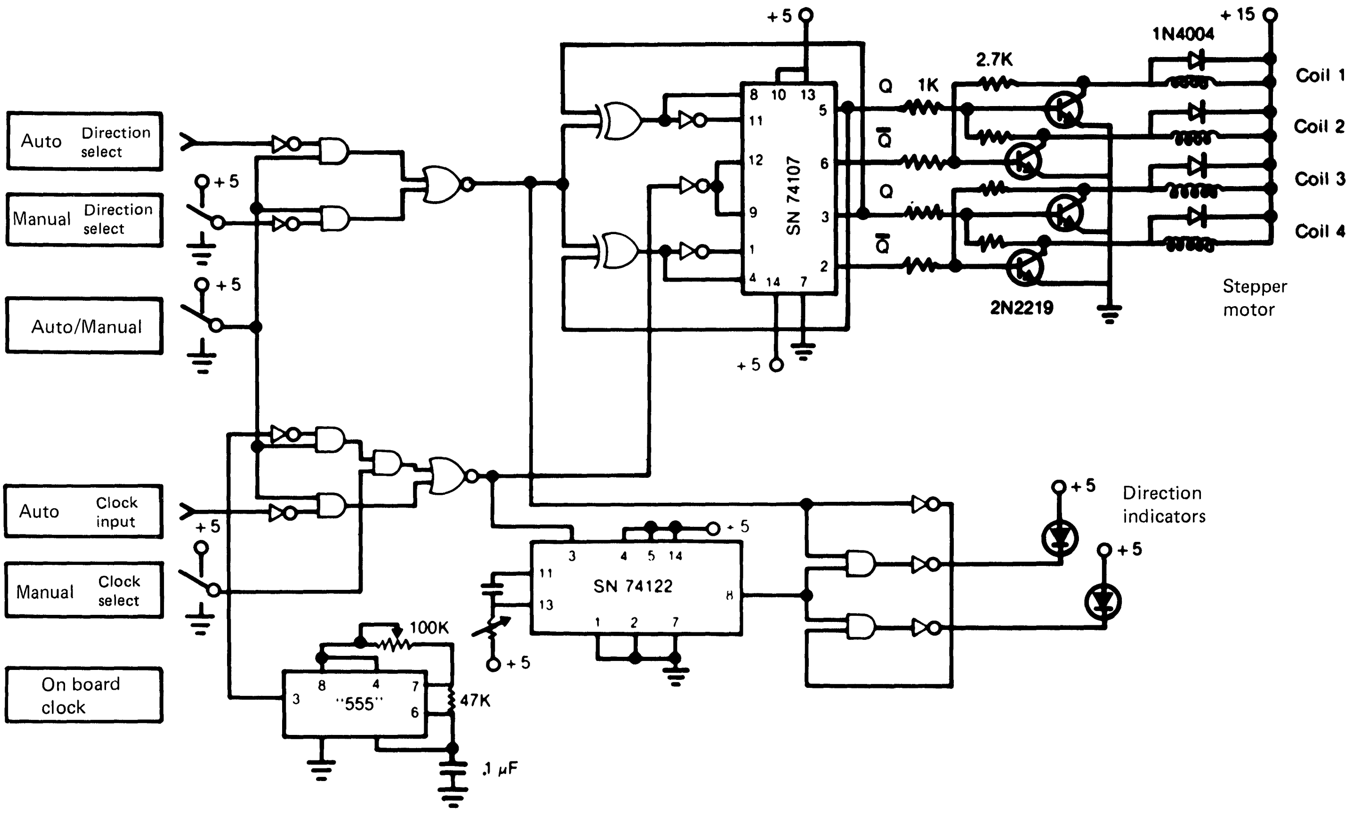

*Figure 4. Electrical schematic of the coolant valve control unit.*

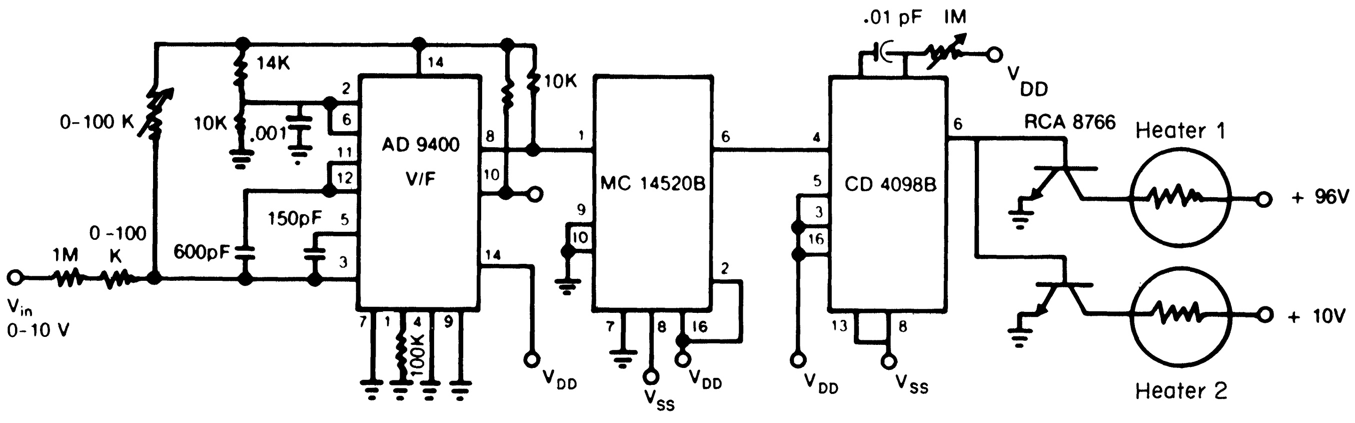

*Figure 5. Electrical schematic of the proportionating heater control circuit.*

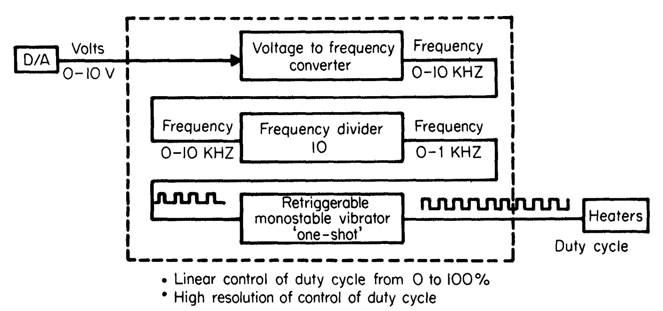

*Figure 6. Functional block diagram of the proportionating heater control circuit.*

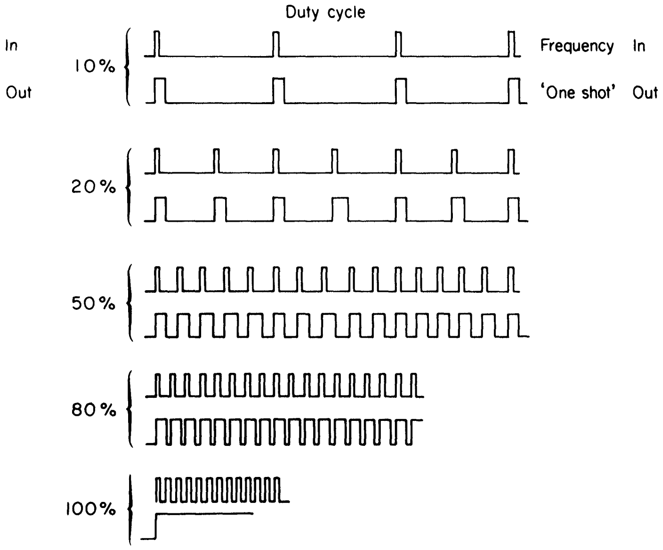

*Figure 7. Proportionating heater control unit input–output signals.*

input, this circuit produces a half on/half off TTL pulse stream. The output of the 'one-shot' is tied to the base of each power transistor and, therefore, directly modulates the flow of current to the heating elements. The resolution of control (0·125 Hz, 0·00125%), fixed by the resolution of the 12-bit D/A interface (1·25 mV), is more than adequate for the requirements outlined above.

### Automation software

The automated chemical synthesis system is controlled by a dedicated MINC LSI 11/2 computer equipped with digital output [2], digital input, preamplifier, A/D, D/A and programmable clock interfaces. The RT11 V4 operating system was selected in order to gain access to the extensive scientific subroutine packages provided by the computer vendor. The automation software is written primarily in FORTRAN with REAL-11/MNC extensions. Copies of the bath-control source code are available from the authors. The bath start-up and temperature change programs are flowcharted (figures 8 and 9) to illustrate the fundamental structure of the automation software for this part of the synthesis system.

### Acknowledgements

The authors wish to thank Robert O. Parker for helpful discussions on heat transfer and temperature control theory.

### References

1. Chodosh, D. F., Wdzieczkowski, F. E., Schainbaum, J. and Berkoff, C. E., *Journal of Automatic Chemistry*, 3 (1983), 99.
2. Winicov, H., Schainbaum, J., Buckley, Jr., J. T., Longino, G., Hill, J. and Berkoff, C. E., *Analytica Chimica Acta*, **103** (1978), 469.
3. Chodosh, D. F., Winicov, H., Buckley, Jr., J. T. and Berkoff, C. E., Computers at the bench (Belgian Pharmaceutical Society International Conference on Computers in Pharmaceutical Research, Namur, Belgium, November 1979).
4. Chodosh, D. F., 1979 Fall D.E.C.U.S. Symposium, Workshop on Laboratory Automation (San Francisco, November 1978).
5. Chodosh, D. F., Buckley, Jr., J. T., Longino, G., Schainbaum, J., Wdzieckzowski, F. E., Winicov, H. and Berkoff, C. E., MINC interfacing (1981 Fall D.E.C.U.S. Symposium, Los Angeles, December 1981).

6. Chodosh, D. F., Buckley, Jr., J. T., Levinson, S. H., Wdzieczkowski, F. E., Weber, J. L., Winicov, H. and Berkoff, C. E., Automated synthesis: design of the chemical reactor hardware (1981 Fall D.E.C.U.S. Symposium, Los Angeles, December 1981).
7. Deming, S. N. and Morgan, S. L. *Analytical Chemistry*, **45** (1973), 278A.
8. Hendrix, C., *Chem. Tech.* (1980), 448.
9. Deming, S. N., *American Laboratory* (1981), 42.

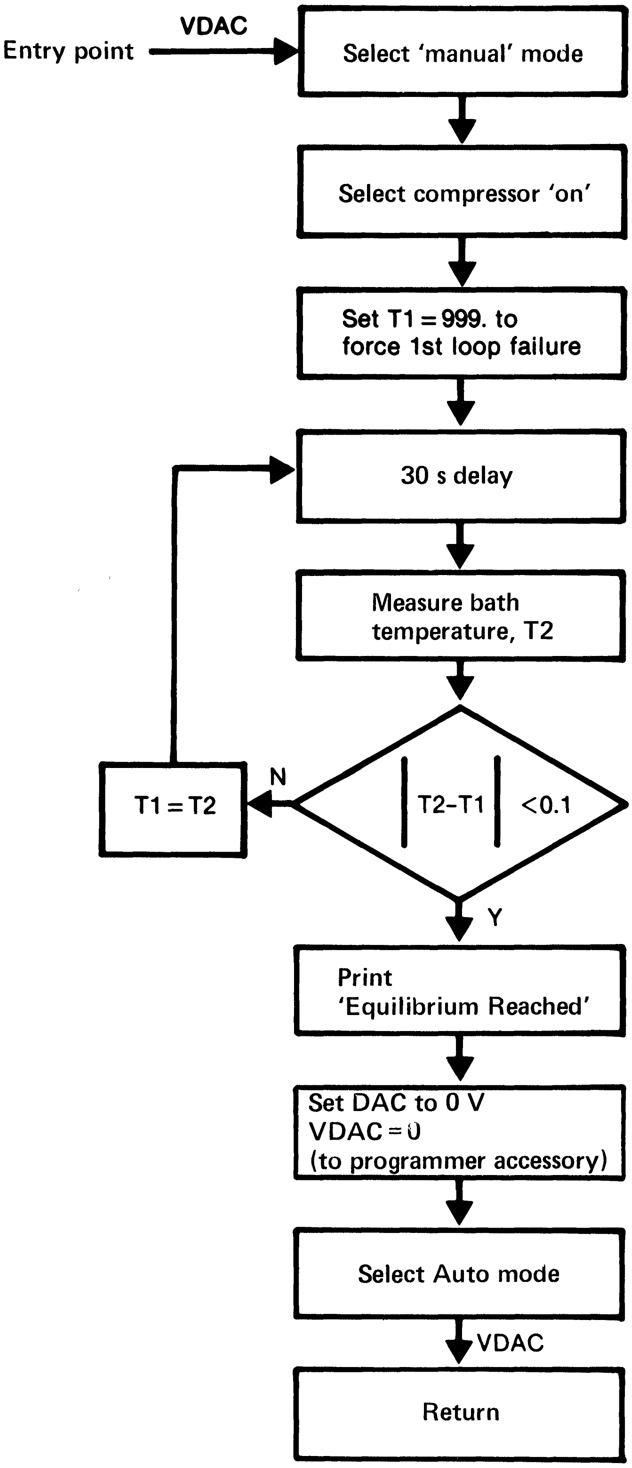

*Figure 8. Flowchart of bath start-up subroutine.*

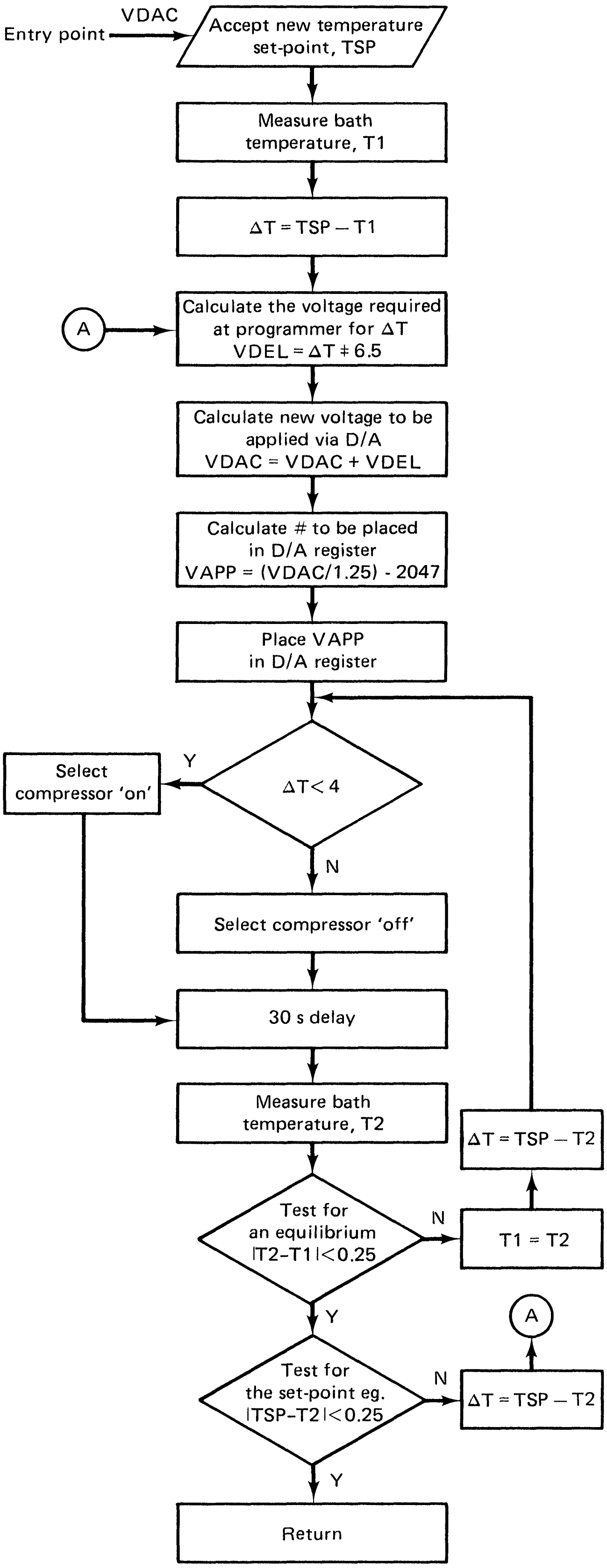

*Figure 9. Flowchart of bath temperature change subroutine (5° < set-point < 35°).*

